# Molecular Identification of *Gambierdiscus* and *Fukuyoa* (Dinophyceae) from Environmental Samples

**DOI:** 10.3390/md15080243

**Published:** 2017-08-02

**Authors:** Kirsty F. Smith, Laura Biessy, Phoebe A. Argyle, Tom Trnski, Tuikolongahau Halafihi, Lesley L. Rhodes

**Affiliations:** 1 Coastal & Freshwater Group, Cawthron Institute, Private Bag 2, 98 Halifax Street East, Nelson 7042, New Zealand; laura.biessy@cawthron.org.nz (L.B.); phoebe.argyle@cawthron.org.nz (P.A.A.); lesley.rhodes@cawthron.org.nz (L.L.R.); 2 School of Biological Sciences, University of Canterbury, Private Bag 4800, 20 Kirkwood Avenue, Christchurch 8041, New Zealand; 3 Auckland War Memorial Museum, Private Bag 92018, Victoria Street West, Auckland 1142, New Zealand; ttrnski@aucklandmuseum.com; 4 Ministry of Fisheries, P.O. Box 871, Nuku’alofa, Tongatapu, Tonga; supi64t@gmail.com

**Keywords:** Quantitative PCR (QPCR), high-throughput sequencing metabarcoding, benthic dinoflagellates, Kermadec Islands, New Zealand, Kingdom of Tonga

## Abstract

Ciguatera Fish Poisoning (CFP) is increasing across the Pacific and the distribution of the causative dinoflagellates appears to be expanding. Subtle differences in thecal plate morphology are used to distinguish dinoflagellate species, which are difficult to determine using light microscopy. For these reasons we sought to develop a Quantitative PCR assay that would detect all species from both *Gambierdiscus* and *Fukuyoa* genera in order to rapidly screen environmental samples for potentially toxic species. Additionally, a specific assay for *F. paulensis* was developed as this species is of concern in New Zealand coastal waters. Using the assays we analyzed 31 samples from three locations around New Zealand and the Kingdom of Tonga. Fourteen samples in total were positive for *Gambierdiscus*/*Fukuyoa* and two samples were also positive using the *F. paulensis* assay. Samples from the Kermadec Islands were further characterized using high-throughput sequencing metabarcoding. The majority of reads corresponded to *Gambierdiscus* species with three species identified at all sites (*G. australes*, *G. honu* and *G. polynesiensis*). This is the first confirmed identification of *G. polynesiensis*, a known ciguatoxin producer, in New Zealand waters. Other known toxin-producing genera were also detected, included *Alexandrium*, *Amphidinium*, *Azadinium*, *Dinophysis*, *Ostreopsis*, and *Prorocentrum*.

## 1. Introduction

The genera *Gambierdiscus* Adachi and Fukuyo and *Fukuyoa* Gómez, Qiu, Lopes and Lin are marine benthic dinoflagellates that are widely distributed from tropical to warm-temperate environments [1,2]. Species in these genera are known to produce the potent neurotoxins ciguatoxin (CTX), and maitotoxin (MTX). CTXs, and potentially MTXs and MTX analogues [3,4,5], accumulate through the food web via uptake by herbivorous fishes and transfer to carnivorous fishes [1]. CFP is associated with human illnesses and even deaths in the Pacific region [6]. Advances in the use of molecular techniques have emphasized the difficulties associated with using morphological characters for the identification of *Gambierdiscus* species and the closely related *Fukuyoa* species. Subtle differences in thecal plate morphology are used to distinguish species which are difficult to view without scanning electron microscopy. This has hindered accurate determination of risk associated with potentially toxic taxa. For example, the original species description of *G. toxicus* [7], the first species linked to CFP [8], included isolates from multiple species, including both toxic and non-toxic species. Studies prior to 2009 describing the toxicity, biology, physiology, and ecology of *G. toxicus* cannot be assigned with any confidence [1,9]. This makes monitoring for potential CFP outbreaks very difficult. For example, the Cook Island Ministry of Marine Resources carries out monthly sampling for potentially toxic dinoflagellates at several lagoon sites around Rarotonga, but *Gambierdiscus* cells can only be identified as *Gambierdiscus* spp. [10] and so estimates of cell concentrations include both toxic and non-toxic species. These types of problems have led to the development of molecular tools for monitoring of *Gambierdiscus* and *Fukuyoa* species. Currently, Quantitative Polymerase Chain Reaction (QPCR) is the most utilized molecular monitoring method as it is cost-effective and rapid [11,12], however, the main limitation of QPCR is that only one to four targets can be detected simultaneously and prior knowledge of the target species is required. 

The genus *Gambierdiscus* currently comprises 15 described species (*G. toxicus* [7]; *G. belizeanus* [13]; *G. australes*, *G. pacificus*, *G. polynesiensis* [14]; *G. caribaeus*, *G. carolinianus*, *G. carpenteri* [9]; *G. excentricus* [15]; *G. scabrosus* [16]; *G. silvae* [17]; *G. balechii* [18]; *G. cheloniae* [19]; *G. honu* [20]; and *G. lapillus* [21]) and six ribotypes (*Gambierdiscus ribotype* 2 [22]; *G.* sp. type 2 [23]; *G.* sp. type 3 [24]; *G.* sp. types 4, 5 and 6 [25]). The genus Fukuyoa currently comprises three described species (*F. yasumotoi* [26]; *F. ruetzleri* [9]; and *F. paulensis* [27]). Several QPCR assays have been developed for the detection of some of these species [11,12]. However, studies are increasingly showing that *Gambierdiscus* and *Fukuyoa* communities consist of multiple co-occurring species [11,25,28]. Therefore, to fully characterise *Gambierdiscus* and *Fukuyoa* communities would currently require at least 25 assays per sample to be performed. Other methods, which detect multiple targets simultaneously, e.g., micro-arrays, are also limited to the detection of specific or predicted targets. With these approaches undescribed species would also go undetected. In order to accurately and fully describe dinoflagellate communities and detect cryptic diversity, the use of high-throughput sequencing (HTS) technologies combined with DNA barcoding methodologies, termed metabarcoding has increased [29,30,31]. HTS metabarcoding uses universal PCR primers to mass-amplify specific gene sequences from environmental samples and enables the characterization of all species or specific taxa present in the sample. This approach allows a greater resolution of microbial community composition than traditional morphological and molecular methodologies. The use of HTS metabarcoding for characterising microbial communities is rapidly increasing due to the adaptability of the methods and a continual lowering of cost per sample [32,33,34].

CFP risk is increasing across the Pacific [3,35], and the distribution of the causative organisms (*Gambierdiscus* species) appears to be expanding, including into more temperate habitats [12,36,37]. Differences in the toxicity of *Gambierdiscus* species and strains also influence the occurrence of CFP and the exact toxin profile most species is unknown [3]. Both *Gambierdiscus* and *Fukuyoa* have been reported from New Zealand’s northern coastal waters [36,38]. The presence of *Gambierdiscus* and *Fukuyoa* in New Zealand indicates a need for pro-active and accurate monitoring of benthic harmful communities in this region [28,29,36,39,40]. In order to rapidly screen environmental samples for potential toxin-producing *Gambierdiscus* and *Fukuyoa* species, we designed a QPCR assay that would detect all species from both genera. Additionally, as *F. paulensis* is known to occur in New Zealand coastal waters, we designed a specific QPCR assay to detect this species. Environmental samples from New Zealand warm-temperate (Northland) and sub-tropical waters (Kermadec Islands) and the Kingdom of Tonga (a tropical site were incidences of CFP occur) were analyzed using both assays. Samples were initially screened using the *Gambierdiscus*/*Fukuyoa* assay and positive samples were subsequently analysed using the *F. paulensis* assay. Samples from the Kermadec Islands were further characterized using dinoflagellate-specific HTS metabarcoding analyses [29].

## 2. Results and Discussion

Due to the difficulties in identifying *Gambierdiscus* and *Fukuyoa* species and the high diversity of species within the genera, including the likelihood of undescribed cryptic taxa, we aimed to develop a single qPCR assay for the rapid detection of all species from both genera. Once environmental samples have been screened using the QPCR assay, the *Gambierdiscus* and *Fukuyoa* community can be further characterized using various molecular tools including species-specific QPCR and HTS metabarcoding. 

**Figure 1:**
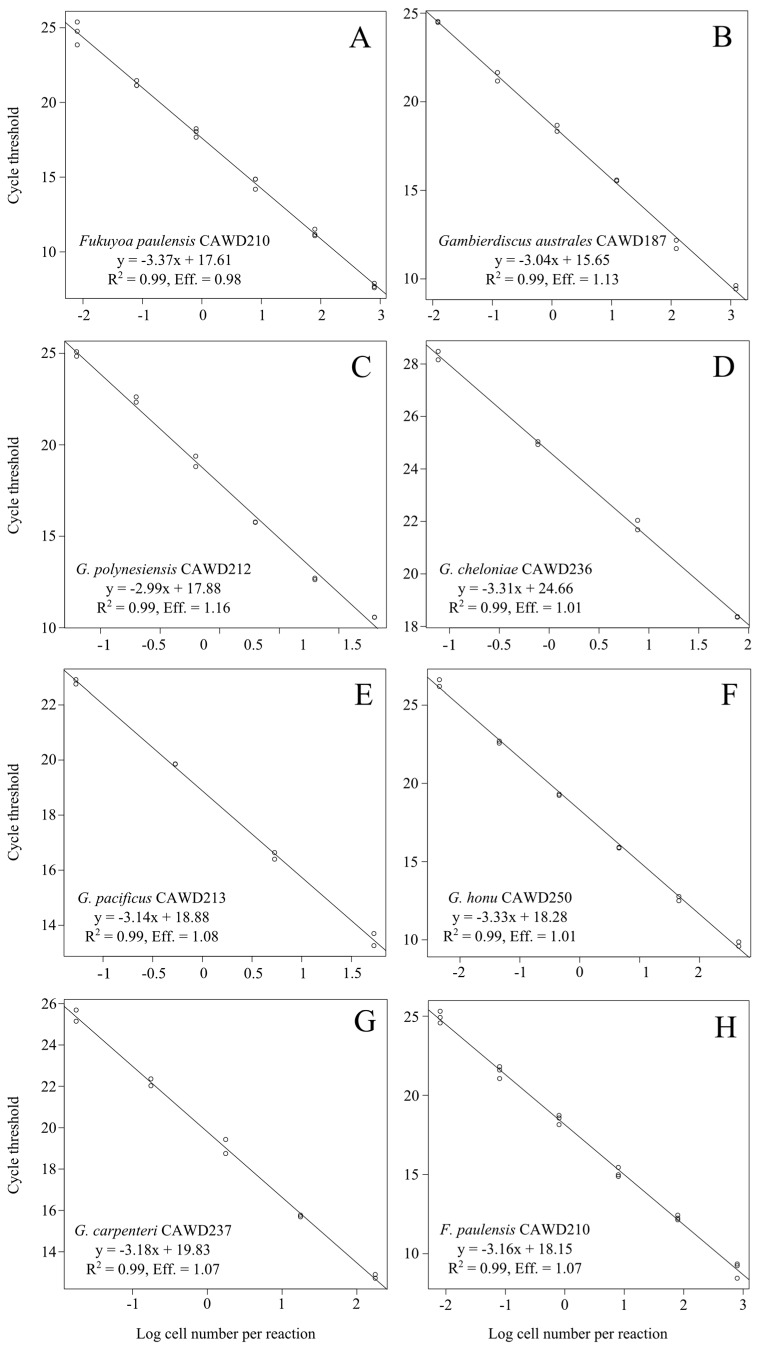
Standard curves for the *Gambierdiscus*/*Fukuyoa* QPCR assay (**A**–**G**) and *Fukuyoa paulensis* QPCR assay (**H**) constructed with 10-fold serial dilutions of genomic DNA extracts from cultures of available *Gambierdiscus* and *Fukuyoa* species.

We also designed a specific QPCR assay for *F. paulensis* as this species is present in New Zealand waters [36]. The QPCR assays designed in this study (Table 1) reliably amplified only the target genera (*Gambierdiscus* and *Fukuyoa*) and species (*F. paulensis*), as determined via cross-reactivity testing with strains listed in Table 2. We were not able to obtain genomic DNA for all known species from the *Gambierdiscus* and *Fukuyoa* genera so were unable to comprehensively assess the assay against all described species. However, *in silico* analyses, using available DNA sequence data for missing species showed the assays are likely to amplify only the specific target genera/species (Figure S1). Both assays had a linear range of detection of six orders of magnitude. The limit of detection was similar for the various species and ranged from approximately 800 cells to well below one cell per reaction (Figure 1). The amplification efficiency of both assays was high when using extracts from cultures of various species as template target (*Gambierdiscus*/*Fukuyoa* assay range: 98–108%; *Fukuyoa paulensis* assay: 107%; Figure 1). 

**Figure 2:**
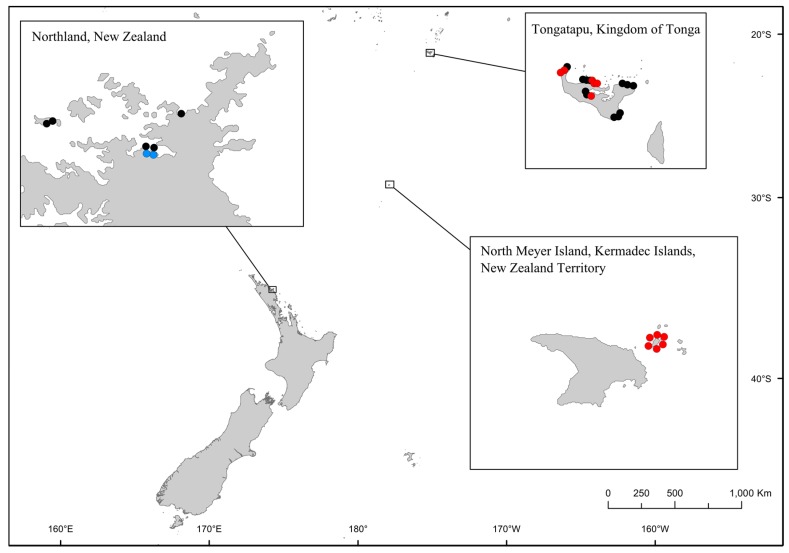
Map of samples sites in New Zealand and the Kingdom of Tonga that were analyzed using the QPCR assays and HTS metabarcoding. Black circles indicate samples that were negative for both QPCR assays, red circles indicate samples that were positive for the *Gambierdiscus*/*Fukuyoa* genus QPCR assay, and blue circles indicate samples that were positive for the *Fukuyoa paulensis*.

Using the *Gambierdiscus* and *Fukuyoa* assay we analyzed 31 samples from three locations: Northland, New Zealand (7 samples), Kermadec Islands, New Zealand Territory (6 samples), and Tonga (18 samples). Samples were initially analyzed using the *Gambierdiscus*/*Fukuyoa* QPCR assay (Figure 2; Table S1). Fourteen samples in total were positive; two from Northland, all six samples from the Kermadec Islands and six samples from Tonga (Figure 2, Table S1). Positive samples were further analyzed using the *F. paulensis* assay. Of these 14 samples, only the two samples from Northland were also positive using the *F. paulensis* assay (Figure 2, Table S1). Concentrations of *F. paulensis* in Te Uenga Bay were low (<1 cell per PCR assay; Figure 2, Table S1). Replicate samples from these sites have previously been analyzed using HTS metabarcoding and *F. paulensis* was also detected in samples from Te Uenga Bay using this method [29]. Morphological surveys have also identified this species in Northland at low cell densities [36].

**Figure 3:**
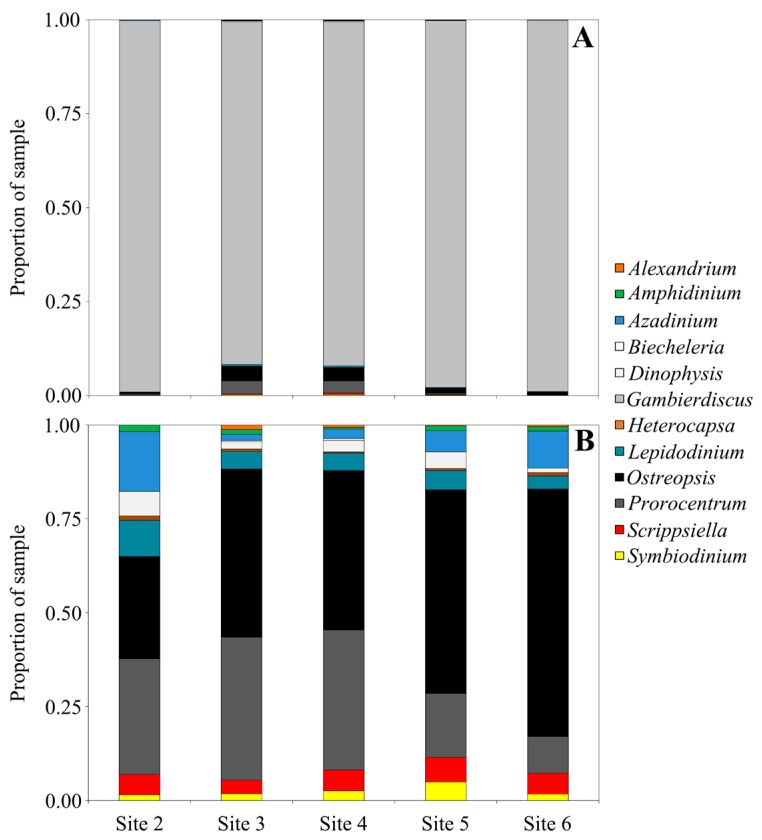
Relative abundance of Dinophyceae reads at genus level from each sampling location at North Meyer Island (Kermadec Islands, New Zealand). (**A**) Includes reads from all genera and (**B**) excludes reads identified as *Gambierdiscus* species.

**Figure 4:**
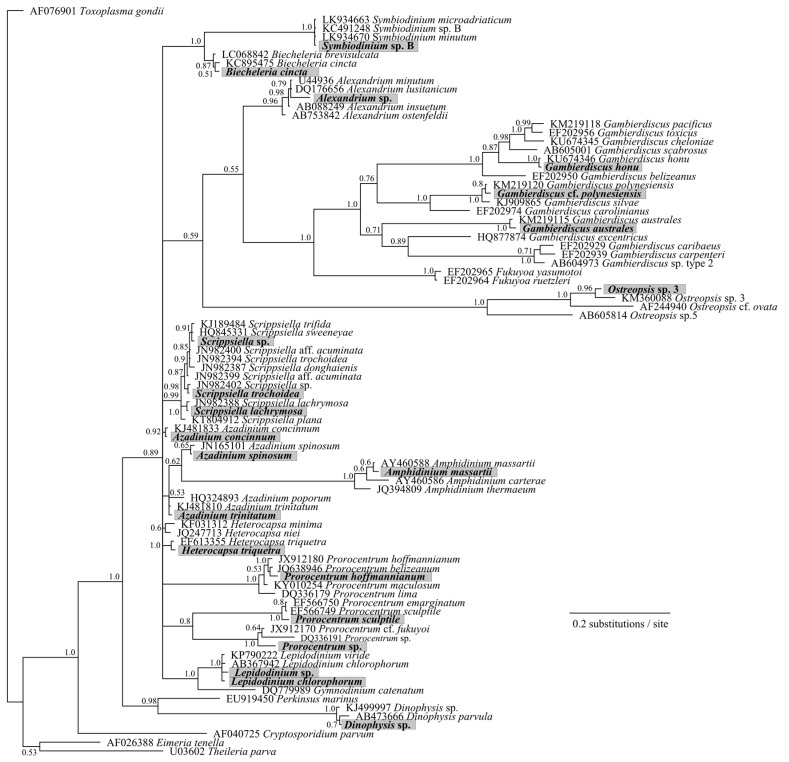
Phylogenetic analysis of large subunit ribosomal RNA (LSU) (D1-D2 region) sequences obtained from the high-throughput sequencing (HTS) metabarcoding using Bayesian analyses. Sequences in bold represent the consensus sequence from all reads of each taxonomic assignment. Values at nodes represent Bayesian posterior probability support. Scale bar is substitutions per site.

**Figure 5:**
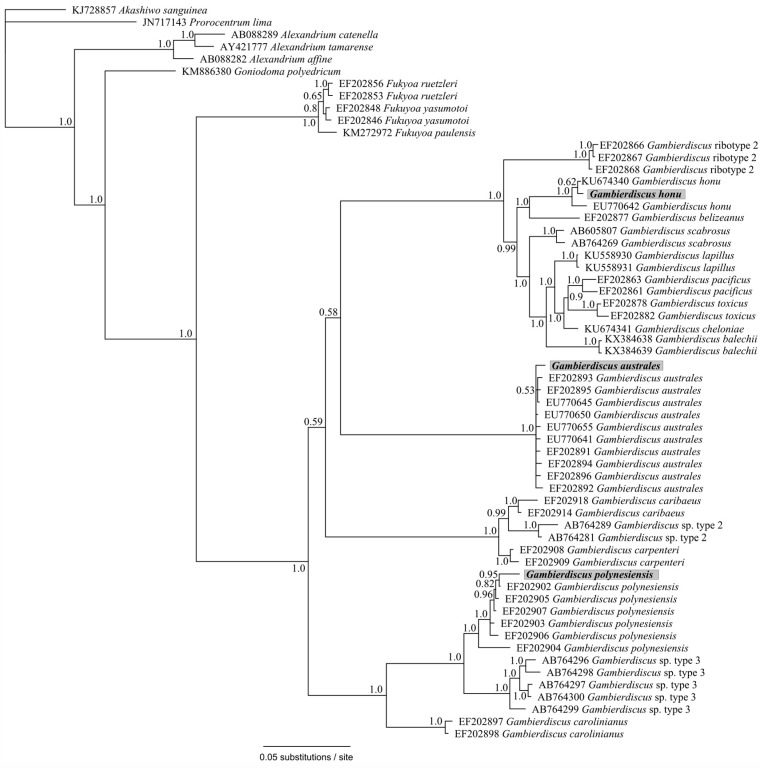
Phylogenetic analysis of partial small subunit ribosomal RNA SSU (V4 region) reads identified as *Gambierdiscus* species obtained from the HTS metabarcoding using Bayesian analyses. Sequences in bold represent the consensus sequence from all reads of each taxonomic assignment. Values at nodes represent Bayesian posterior probability support. Scale bar is substitutions per site.

Samples from North Meyer Island were collected in November 2015 during an expedition to the Kermadec Islands [28]. North Meyer Island is about 2 km north east of Rangitahua (Raoul Island), Kermadec Islands. The samples from North Meyer Island were positive for *Gambierdiscus*/*Fukuyoa* species but not *F. paulensis*. In order to fully characterize the *Gambierdiscus/Fukuyoa* present in the samples HTS metabarcoding was employed. DNA extracts from five of the sites were of sufficient quality and quantity for HTS metabarcoding. Sequencing targeting the LSU D1-D2 region generated between 8693 and 13,210 Dinophyceae reads per sample after quality filtering, size trimming, and chimera detection (Table S2). The dinoflagellate species identified at each site was similar. Site 6 had the lowest number of species (17) while the other four sites had 19–21 species identified. The majority of the reads corresponded to *Gambierdiscus* species (Figure 3A) with three species identified at all sites (*G. australes*, *G. honu* and *G. polynesiensis*; Table S2, Figure 4). *G. australes* was the dominant species, representing over 89% of reads identified at each site. The newly described *G. honu* represented less than 1% of all reads and *G. polynesiensis* represented less than 5% of all reads. *G. polynesiensis* is known to produce CTX, and *G. australes* and *G. honu* produce MTX-1/MTX-3 and MTX-3 respectively [28]. Other known toxin-producing genera detected included *Alexandrium*, *Amphidinium*, *Azadinium*, *Dinophysis*, *Ostreopsis*, and *Prorocentrum*, although sequence reads for these taxa were low (Figure 3A,B and Figure 4, Table S2). Morphological analyses from samples collected during the same survey (albeit not true replicate samples) identified the dinoflagellate species: *G. australes*, *G. honu*, *Amphidinium carterae*, *Coolia malayensis*, *Prorocentrum hoffmannianum*, and *Ostreopsis* sp. 3 [28]. Cells were also tentatively identified as *G. polynesiensis* but did not survive culturing. The HTS metabarcoding analyses also detected these same species with the exceptions of *Amphidinium carterae* and *Coolia malayensis*, indicating that these species may have a patchy distribution in the area. Morphological analyses also confirmed that *Gambierdiscus* cells were present [28]. Previously isolated strains of *Ostreopsis* sp. 3 from the Kermadec Islands (isolate CAWD221) [40] and the Cook Islands (isolate CAWD184) [41] produce low concentrations of palytoxin-like compounds, however the isolate from North Meyer Island (isolate CAWD241) is non-toxic [28]. Planktonic dinoflagellates were also detected in the metabarcoding analyses, including known toxic genera *Alexandrium* and *Azadinium*, presumably present as cysts in the benthos. The symbiotic dinoflagellate, *Symbiodinium*, was also present. In order to confirm the identification of the *Gambierdiscus* species, in particular *G. polynesiensis* as this species has not been detected in New Zealand water previously, we also sequenced amplicons from the SSU V4 region. The consensus sequences from all reads classified as *Gambierdiscus* species were aligned with reference sequences. The identification of *G. polynesiensis*, *G. honu* and *G. australes* was supported by the phylogenetic analyses (Figure 5). The consensus sequence from the *G. polynesiensis* reads clustered within other sequences from *G. polynesiensis* strains, confirming the presence of this species in the samples from the Kermadec Islands (Figure 5). Overall, the genera identified using metabarcoding were similar to previous analyses using metabarcoding in Northland, New Zealand with the exception of *Gambierdiscus* spp. being the dominant taxa in the Kermadec Islands. In Northland, samples were dominated by *Ostreopsis* cf. *siamensis*, and *Fukuyoa paulensis* was present at low concentrations [29].

Phylogenetic analyses showed that all taxa classifications using the custom dinoflagellate sequence databases were correct (Figure 4 and Figure 5). Some species groups were able to be well resolved using the short sequences (approximately 450 bp) such as *Gambierdiscus* spp., *Ostreopsis* spp., and *Amphidinium* spp. (Figure 4). While other groups could not be successfully resolved, for example, *Azadinium* spp. were split and did not cluster all together, and *Symbiodinium* spp./*Lepidodinium* spp. were not able to be differentiated at the species level (Figure 4). Although, the short sequences used to generate the phylogeny were not able to resolve some phylogenetic relationships, the resulting tree did confirm all species designations to be correct. Several classifications could not be identified to the species level (e.g., *Alexandrium* sp., *Prorocentrum* sp., and *Dinophysis* sp.) either because they represent undescribed species or species that are not present in GenBank database.

*Gambierdiscus*/*Fukuyoa* species were detected in six of the eighteen samples collected from Tonga. Unfortunately, these samples were not able to be fully characterized using HTS metabarcoding as DNA quantity was low. Rates of CFP have increased in Tonga over the last four decades [35] but little is known about the diversity, abundance or toxicity of benthic dinoflagellate species in this region. Further surveys of the *Gambierdiscus*/*Fukuyoa* communities in Tonga, including morphological and molecular descriptions, are currently being undertaken (P. Argyle, unpubl. data).

## 3. Materials and Methods 

The target positions for forward and reverse primers were designed using multiple alignments of the large subunit ribosomal RNA (LSU) and the ribosomal internal transcribed spacer (ITS) region (ClustalW) [42] for the *Gambierdiscus*/*Fukuyoa* genus and *Fukuyoa paulensis* assays respectively. Sequences from *Gambierdiscus* and *Fukuyoa* species, and other Gonyaulacales dinoflagellates obtained from GenBank (http://www.ncbi.nlm.nih.gov/genbank/). The specificity of the primer sequences was then confirmed using BLAST (Basic Local Alignment Search Tool) at NCBI (National Centre for Biotechnology Information). The assay was optimized on a Rotor-Gene 6000 (Corbett, Sydney, NSW, Australia), using genomic DNA extracted from an exponentially growing culture of *F. paulensis* (strain CAWD210) isolated from Northland, New Zealand [36]. Genomic DNA was extracted using PowerSoil^®^ DNA isolation kits (Mo Bio, Carlsbad, CA, USA) following the manufacturer’s instructions. The optimized assays consisted of a 20 μL reaction containing 10 μL of Platinum^®^ SYBR^®^ Green QPCR SuperMix-UDG (Invitrogen, Carlsbad, CA, USA), 1 μM of each primer, 0.8 μg non-acetylated bovine serum albumin (BSA; Sigma-Aldrich, Auckland, New Zealand), and 10 ng of DNA template. Primer sequences for each assay are shown in Table 1.

All PCR reactions in this study were set up manually and all included both positive and no template control reactions. Positive controls were 10 ng of DNA from an extract of an *F. paulensis* (strain CAWD210) culture and no template controls contained 2 μL of sterile water as a substitute for DNA template. Assays were run in clear 0.2 mL thin-wall PCR tubes (Axygen, Union City, CA, USA). PCR cycling conditions were: 50 °C for 2 min, 95 °C for 2 min and 30 cycles of 95 °C for 15 s and 62 °C for 60 s. The specificity of the assays was verified using DNA from various *Gambierdiscus* and *Fukuyoa* species, and other related species (Table 2). DNA from each species (10 ng) was used in the QPCR assays as described above. The amplification efficiency of the assay was determined by using 10-fold serially-diluted DNA extracts from various available cultures of *Gambierdiscus* and *Fukuyoa* with known cell concentration. The amplification efficiency was calculated from the slopes of the regression curve derived from the standard curve. Three separate DNA extracts were analyzed in triplicate and ranged from approximately 800 to 0.004 cells per reaction.

Environmental samples were collected from the Bay of Islands, Northland, New Zealand (see Smith et al. for more information on sampling locations [29]), North Meyer Island, Kermadec Islands, New Zealand (see Rhodes et al. for more information on sampling locations [28]), and Tongatapu, Kingdom of Tonga (Figure 1 and Figure 2, Table S1). To target benthic-epiphytic dinoflagellates, dominant macro-algal species at each site were collected. Approximately 500 g of mixed, submerged macro-algal species was pooled in clean plastic bags with 500 mL of ambient seawater. Bags were gently shaken to dislodge dinoflagellates and subsamples (500 mL) were then collected in containers and left to settle for eight hours, after which the supernatant was carefully decanted. Preservation solution for nucleic acids [43] was added (40 mL) to the remaining sediment slurry (approximately 10 mL) and the samples stored at ambient temperature until DNA extraction. Genome DNA was extracted as described above. The DNA samples were then analysed using the *Gambierdiscus*/*Fukuyoa* QPCR assay. Reactions were deemed positive if an increase in fluorescence was detected before 30 cycles and the melt temperature of the product was between 83 °C and 88 °C. Positive samples were then analyzed using the *Fukuyoa paulensis* QPCR assays.

In order to fully characterize the *Gambierdiscus* species present in the Kermadec Islands duplicate DNA extractions were also amplified using primer pairs targeting the D1-D2 region of the LSU and the V4 region of the SSU for high-throughput sequencing (HTS) metabarcoding [29]. Samples were amplified using primers from Smith et al. (2017) (Table 1). Samples from Northland were previously analyzed using metabarocoding [29] and the DNA extractions from Tonga were not suitable for metabarcoding. The primers were modified to include Illumina^TM^ overhang adaptors. DNA extracts were sent to New Zealand Genomics Limited (NZGL, Massey Genome Service, Massey University, Palmerston North, New Zealand) for further processing. Samples were normalized to five ng·uL^−1^. Libraries were prepared using the Illumina^TM^ two-step PCR amplicon library preparation method and sequenced using an Illumina^TM^ MiSeq sequencer with 2X 250 base paired-end reads. All data generated was quality checked by NZGL using the following processes; FastQC, FastQscreen, and SolexaQA [44]. Further analyses were carried out in Mothur v1.37.6 [45] as described previously [29]. Bayesian analyses were carried out in Geneious^®^ using MrBayes 3.1.2 [46]. The evolutionary model (general time reversible with gamma-shaped among-site variation, GTR+G) was selected using MrModeltest v 2.2 [47]. The consensus sequences from all reads of each taxonomic assignment were aligned with references sequences. Analyses of alignments were carried out in two simultaneous runs with four chains each 2 × 10^6^ generations, sampling every 1000 trees, discarding a burn-in period of the first 1000 sampling points. After 2 × 10^6^ generations, potential scale reduction factor values were approximately 1.0 and average standard deviation of split frequencies were less than 0.01.

## 4. Conclusions

The QPCR assays developed in this study demonstrated high specificity and sensitivity. The assays allowed rapid determination of the presence of *Gambierdiscus*/*Fukuyoa* species in environmental samples, enabling select samples to be targeted for further characterization. It is also an advantageous method for monitoring risk at sites where cell concentrations extremely low (e.g., Northland, New Zealand). HTS metabarcoding (targeting the D1-D2 region of the LSU and the V4 region of the SSU) was used to identify all the co-occurring *Gambierdiscus* species from the Kermadec samples, as well as the full dinoflagellate community. This is the first confirmed identification of *G. polynesiensis*, a species associated with CFP, in New Zealand waters. In a more recent sampling expedition to the nearby North Meyer Island (part of the Kermadec archipelago) *G. polynesiensis* cells were isolated and characterized [48]. The presence of this *G. polynesiensis* in New Zealand warrants further investigation as this species can pose a serious risk to human health via the food chain. HTS metabarcoding shows huge potential for characterizing dinoflagellate communities to species-level resolution but turn-around times and costs are still significant, and currently metabarcoding is not suitable for routine monitoring purposes. However, rapid advances in HTS technologies mean these limitations will reduce in the future. At present, the *Gambierdiscus*/*Fukuyoa* genus-level QPCR assay is an extremely useful tool for monitoring programmes and taxonomic surveys worldwide. By screening environmental samples for the presence of these potentially toxic taxa, sites with potential risk can be rapidly identified and prioritized for more in-depth species-level characterization.

## Figures and Tables

**Table 1:** Sequences of primers used in this study.

Target	Sequence	Gene Region	Product Size	Type
*Gambierdiscus*/*Fukuyoa*				
GA668-F	5′-CTGTRTGACCCGTCTTGAAAC-3′	D2-D3 LSU	208 bp	QPCR
GA875-R	5′-GTTTCCCCTGVCTTGRCC-3′			
*Fukuyoa paulensis*				
FP128-F	5′-AAAGTGGGAAAAGGGTGG-3′	ITS	128 bp	QPCR
FP253-R	5′-TGTCACCGCACCAAAATC-3′			
Dinoflagellates				
D1R	5′-ACCCGCTGAATTTAAGCATA-3′	D1-D2 LSU	365 bp	HTS Metabarcoding
305R	5′-TTTAAYTCTCTTTYCAAAGTCC-3′			
Dinoflagellates				
SSU556F	5′-CGCGGTAATTCCAGCTYC-3′	V4 SSU	346 bp	HTS Metabarcoding
SSU911R	5′-ATYCAAGAATTTCACCTCTGAC-3′			

**Table 2:** Strains used for cross-reactivity testing of the *Gambierdiscus*/*Fukuyoa* QPCR assays. Results show either positive (+) or negative (−) for the duplicate PCR assays. NT, not tested. NA, not applicable. Temp., Temperature (°C).

Species	Strain	*Gambierdiscus*/*Fukuyoa*		*Fukuyoa paulensis*	
		Result	Melt Temp.	Result	Melt Temp.
*Gambierdiscus pacificus*	CAWD213	+/+	86.0	−/−	NA
*Gambierdiscus australes*	CAWD149	+/+	86.0	−/−	NA
*Gambierdiscus polynesiensis*	CAWD212	+/+	85.5	−/−	NA
*Gambierdiscus cheloniae*	CAWD232	+/+	85.8	−/−	NA
*Gambierdiscus honu*	CAWD233	+/+	85.5	−/−	NA
*Gambierdiscus* ribotype II	CCMP1655	+/+	83.5	−/−	NA
*Gambierdiscus caribaeus*	CCMP1733	+/+	85.5	−/−	NA
*Gambierdiscus carpenteri*	CCMP1654	+/+	84.5	−/−	NA
*Gambierdiscus belizeanus*	CCMP401	+/+	85.7	−/−	NA
*Gambierdiscus carolinianus*	Kenny6	+/+	87.8	−/−	NA
*Fukuyoa ruetzleri*	Gam1	+/+	84.0	−/−	NA
*Fukuyoa paulensis*	CAWD210	+/+	83.3	+/+	82.0
*Fukuyoa paulensis*	CAWD211	NT	NT	+/+	81.8
*Ostreopsis* sp.	CAWD239	−/−	NA	−/−	NA
*Ostreopsis* sp.	L138	−/−	NA	−/−	NA
*Coolia malayensis*	L130	−/−	NA	−/−	NA
*Alexandrium margalefii*	K53	−/−	NA	−/−	NA
*Alexandrium catenella*	K54	−/−	NA	−/−	NA
*Prorocentrum* cf. *micans*	L144	−/−	NA	−/−	NA
*Karenia mikimotoi*	CAWD63	−/−	NA	−/−	NA
*Gymnodinium impudicum*	CAWD139	−/−	NA	−/−	NA
*Amphidinium massartii*	L132	−/−	NA	−/−	NA

## Supplementary Materials

The following are available online at www.mdpi.com/1660-3397/15/8/243/s1, Figure S1: Alignments (ClustalW; [42]) of available DNA sequences of *Gambierdiscus* and *Fukuyoa* species not able to be directly tested, and primers for both the (A) *Gambierdiscus*/*Fukuyoa* and (B) *Fukuyoa paulensis* QPCR assays. Table S1: QPCR assay results for field samples collected in New Zealand and the Kingdom of Tonga. NT, not tested. Table S2: Average read number and classification levels at 98% identity for the LSU gene region from each sampling site from North Meyer Island, Kermadec Islands, New Zealand.

## References

[1] Parsons M.L., Aligizaki K., Bottein M.Y.D., Fraga S., Morton S.L., Penna A., Rhodes L. (2012). *Gambierdiscus* and *Ostreopsis*: Reassessment of the state of knowledge of their taxonomy, geography, ecophysiology, and toxicology. Harmful Algae.

[2] Hoppenrath M., Murray S.A., Chomerat N., Horiguchi T. (2014). Marine Benthic Dinoflagellates—Unveiling Their Worldwide Biodiversity.

[3] Kohli G.S., Farrell H., Murray S.A., Botana L.M., Louzao C., Vilarino N. (2015). *Gambierdiscus*, the cause of ciguatera fish poisoning: An increased human health threat influenced by climate change. Climate Change and Marine and Freshwater Toxins.

[4] Laza-Martínez A., David H., Riobó P., Miguel I., Orive E. (2016). Characterization of a strain of *Fukuyoa paulensis* (Dinophyceae) from the Western Mediterranean Sea. J. Eukaryot. Microbiol..

[5] Kohli G.S., Papiol G.G., Rhodes L.L., Harwood D.T., Selwood A., Jerrett A., Murray S.A., Neilan B.A. (2014). A feeding study to probe the uptake of maitotoxin by snapper (*Pagrus auratus*). Harmful Algae.

[6] Berdalet E., Bravo I., Evans J., Fraga S., Kibler S., Kudela M., Larsen J., Litaker W., Penna A., Tester P. (2012). Global Ecology and Oceanography of Harmful Algal Blooms, GEOHAB Core Research Project: HABs in Benthic Systems.

[7] Adachi R., Fukuyo Y. (1979). The thecal structure of a marine toxic dinoflagellate *Gambierdiscus toxicus* gen. et sp. nov. collected in a Ciguatera-endemic area. Bull. Jpn. Soc. Sci. Fish..

[8] Bagnis R., Chanteau S., Chungue E., Hurtel J.M., Yasumoto T., Inoue A. (1980). Origins of Ciguatera Fish Poisoning: A new dinoflagellate, *Gambierdiscus toxicus* Adachi and Fukuyo, definitively involved as a causal agent. Toxicon.

[9] Litaker R.W., Vandersea M.W., Faust M.A., Kibler S.R., Chinain M., Holmes M.J., Holland W.C., Tester P.A. (2009). Taxonomy of G*ambierdiscus* including four new species, *Gambierdiscus caribaeus*, *Gambierdiscus carolinianus*, *Gambierdiscus carpenteri* and *Gambierdiscus ruetzleri* (Gonyaulacales, Dinophyceae). Phycologia.

[10] Rongo T., van Woesik R. (2012). Socioeconomic consequences of Ciguatera poisoning in Rarotonga, Southern Cook Islands. Harmful Algae.

[11] Vandersea M.W., Kibler S.R., Holland W.C., Tester P.A., Schultz T.F., Faust M.A., Holmes M.J., Chinain M., Litaker R.W. (2012). Development of semi-quantitative pcr assays for the detection and enumeration of *Gambierdiscis* species (Gonyaulacales, Dinophyceae). J. Phycol..

[12] Nishimura T., Hariganeya N., Tawong W., Sakanari H., Yamaguchi H., Adachi M. (2016). Quantitative PCR assay for detection and enumeration of Ciguatera-causing dinoflagellate *Gambierdiscus* spp. (Gonyaulacales) in coastal areas of japan. Harmful Algae.

[13] Faust M.A. (1995). Observation of sand-dwelling toxic dinoflagellates (Dinophyceae) from widely differing sites, including two new species. J. Phycol..

[14] Chinain M., Faust M.A., Pauillac S. (1999). Morphology and molecular analyses of three toxic species of *Gambierdiscus* (Dinophyceae): *G. pacificus*, sp. nov., *G. australes*, sp. nov., and *G. polynesiensis*, sp. nov.. J. Phycol..

[15] Fraga S., Rodríguez F., Caillaud A., Diogène J., Raho N., Zapata M. (2011). *Gambierdiscus excentricus* sp. nov. (Dinophyceae), a benthic toxic dinoflagellate from the Canary Islands (NE Atlantic Ocean). Harmful Algae.

[16] Nishimura T., Sato S., Tawong W., Sakanari H., Yamaguchi H., Adachi M. (2014). Morphology of *Gambierdiscus scabrosus* sp. Nov. (Gonyaulacales): A new epiphytic toxic dinoflagellate from coastal areas of Japan. J. Phycol..

[17] Fraga S., Rodríguez F. (2014). Genus *Gambierdiscus* in the Canary Islands (NE atlantic ocean) with description of *Gambierdiscus silvae* sp. nov., a new potentially toxic epiphytic benthic dinoflagellate. Protist.

[18] Fraga S., Rodríguez F., Riobó P., Bravo I. (2016). *Gambierdiscus balechii* sp. nov (Dinophyceae), a new benthic toxic dinoflagellate from the Celebes Sea (SW Pacific Ocean). Harmful Algae.

[19] Smith K.F., Rhodes L., Verma A., Curley B.G., Harwood D.T., Kohli G.S., Solomona D., Rongo T., Munday R., Murray S.A. (2016). A new *Gambierdiscus* species (Dinophyceae) from Rarotonga, Cook Islands: *Gambierdiscus cheloniae* sp. nov.. Harmful Algae.

[20] Rhodes L., Smith K.F., Verma A., Curley B.G., Harwood D.T., Murray S., Kohli G.S., Solomona D., Rongo T., Munday R. (2017). A new species of *Gambierdiscus* (Dinophyceae) from the South-West Pacific: *Gambierdiscus honu* sp. nov.. Harmful Algae.

[21] Kretzschmar A.L., Verma A., Harwood T., Hoppenrath M., Murray S. (2017). Characterization of *Gambierdiscus lapillus* sp. nov. (Gonyaulacales, Dinophyceae): A new toxic dinoflagellate from the Great Barrier Reef (Australia). J. Phycol..

[22] Litaker R.W., Vandersea M.W., Faust M.A., Kibler S.R., Nau A.W., Holland W.C., Chinain M., Holmes M.J., Tester P.A. (2010). Global distribution of Ciguatera causing dinoflagellates in the genus *Gambierdiscus*. Toxicon.

[23] Kuno S., Kamikawa R., Yoshimatsu S., Sagara T., Nishio S., Sako Y. (2010). Genetic diversity of *Gambierdiscus* spp. (Gonyaulacales, Dinophyceae) in Japanese coastal areas. Phycol. Res..

[24] Nishimura T., Sato S., Tawong W., Sakanari H., Uehara K., Shah M.M.R., Suda S., Yasumoto T., Taira Y., Yamaguchi H. (2013). Genetic diversity and distribution of the Ciguatera-causing dinoflagellate *Gambierdiscus* spp. (Dinophyceae) in coastal areas of Japan. PLoS ONE.

[25] Xu Y., Richlen M.L., Morton S.L., Mak Y.L., Chan L.L., Tekiau A., Anderson D.M. (2014). Distribution, abundance and diversity of *Gambierdiscus* spp. from a Ciguatera-endemic area in Marakei, republic of Kiribati. Harmful Algae.

[26] Holmes M.J. (1998). *Gambierdiscus yasumotoi* sp. nov. (Dinophyceae), a toxic benthic dinoflagellate from Southeastern Asia. J. Phycol..

[27] Gómez F., Qiu D., Lopes R.M., Lin S. (2015). *Fukuyoa paulensis* gen. et sp. nov., a new genus for the globular species of the dinoflagellate G*ambierdiscus* (Dinophyceae). PLoS ONE.

[28] Rhodes L.L., Smith K.F., Verma A., Murray S., Harwood D.T., Trnski T. (2017). The dinoflagellate genera *Gambierdiscus* and *Ostreopsis* from subtropical Raoul Island and North Meyer Island, Kermadec Islands. N. Z. J. Mar. Freshw. Res..

[29] Smith K.F., Kohli G.S., Murray S.A., Rhodes L.L. (2017). Assessment of the metabarcoding approach for community analysis of benthic-epiphytic dinoflagellates using mock communities. N. Z. J. Mar. Freshw. Res..

[30] Kohli G.S., Neilan B.A., Brown M.V., Hoppenrath M., Murray S.A. (2013). Cob gene pyrosequencing enables characterization of benthic dinoflagellate diversity and biogeography. Environ. Microbiol..

[31] Le Bescot N., Mahé F., Audic S., Dimier C., Garet M.-J., Poulain J., Wincker P., Vargas C., Siano R. (2015). Global patterns of pelagic dinoflagellate diversity across protist size classes unveiled by metabarcoding. Environ. Microbiol..

[32] Bik H.M., Porazinska D.L., Creer S., Caporaso J.G., Knight R., Thomas W.K. (2012). Sequencing our way towards understanding global eukaryotic biodiversity. Trends Ecol. Evol..

[33] Lallias D., Hiddink J.G., Fonseca V.G., Gaspar J.M., Sung W., Neill S.P., Barnes N., Ferrero T., Hall N., Lambshead P.J.D. (2015). Environmental metabarcoding reveals heterogeneous drivers of microbial eukaryote diversity in contrasting estuarine ecosystems. ISME J..

[34] Massana R., Gobet A., Audic S., Bass D., Bittner L., Boutte C., Chambouvet A., Christen R., Claverie J.-M., Decelle J. (2015). Marine protist diversity in European coastal waters and sediments as revealed by high-throughput sequencing. Environ. Microbiol..

[35] Skinner M.P., Brewer T.D., Johnstone R., Fleming L.E., Lewis R.J. (2011). Ciguatera fish poisoning in the Pacific Islands (1998 to 2008). PLoS Negl. Trop. Dis..

[36] Rhodes L., Papiol G.G., Smith K., Harwood T. (2014). *Gambierdiscus* cf. *yasumotoi* (Dinophyceae) isolated from new zealand’s sub-tropical northern coastal waters. N. Z. J. Mar. Freshw. Res..

[37] Kohli G.S., Murray S.A., Neilan B.A., Rhodes L.L., Harwood D.T., Smith K.F., Meyer L., Capper A., Brett S., Hallegraeff G.M. (2014). High abundance of the potentially maitotoxic dinoflagellate *Gambierdiscus carpenteri* in temperate waters of new south wales, australia. Harmful Algae.

[38] Chang F.H. (1996). Shellfish toxin update. Seaf. N. Z..

[39] Rhodes L., Smith K., Papiol G.G., Adamson J., Harwood T., Munday R. (2014). Epiphytic dinoflagellates in sub-tropical New Zealand, in particular the genus *Coolia* Meunier. Harmful Algae.

[40] Rhodes L., Smith K., Harwood T., Bedford C. (2014). Novel and toxin-producing epiphytic dinoflagellates isolated from sub-tropical Raoul Island, Kermadec Islands group. N. Z. J. Mar. Freshw. Res..

[41] Selwood A.I., van Ginkel R., Harwood D.T., McNabb P.S., Rhodes L.R., Holland P.T. (2012). A sensitive assay for palytoxins, ovatoxins and ostreocins using LC-MS/MS analysis of cleavage fragments from micro-scale oxidation. Toxicon.

[42] Thompson J.D., Higgins D.G., Gibson T.J. (1994). Clustal W: Improving the sensitivity of progressive multiple sequence alignment through sequence weighting, position-specific gap penalties and weight matrix choice. Nucleic Acids Res..

[43] De Wit P., Pespeni M.H., Ladner J.T., Barshis D.J., Seneca F., Jaris H., Therkildsen N.O., Morikawa M., Palumbi S.R. (2012). The simple fool's guide to population genomics via RNA-seq: An introduction to high-throughput sequencing data analysis. Mol. Ecol. Resour..

[44] Cox M., Peterson D., Biggs P. (2010). SolexaQA: At-a-glance quality assessment of illumina second-generation sequencing data. BMC Bioinform..

[45] Schloss P.D., Westcott S.L., Ryabin T., Hall J.R., Hartmann M., Hollister E.B., Lesniewski R.A., Oakley B.B., Parks D.H., Robinson C.J. (2009). Introducing MOTHUR: Open-source, platform-independent, community-supported software for describing and comparing microbial communities. Appl. Environ. Microbiol..

[46] Huelsenbeck J.P., Ronquist F. (2001). MrBayes: Bayesian inference of phylogenetic trees. Bioinformatics.

[47] Nylander J.A.A. (2004). Mrmodeltest V2 Program Distributed by the Author.

[48] Rhodes L.L., Smith K.F., Murray S., Harwood D.T., Trnski T., Munday M. (2017). The epiphytic genus *Gambierdiscus* (Dinophyceae) in the Kermadec Islands and Zealandia regions of the southwestern Pacific and the associated risk of ciguatera fish poisoning. Mar. Drugs.

