# Supplementary material for: Molecular Identification of Gambierdiscus and Fukuyoa (Dinophyceae) from Environmental Samples
Source: Mar Drugs. 2017 Aug 2;15(8):243. doi: 10.3390/md15080243 (PMC5577598; doi:10.3390/md15080243)
Supplement: Supplementary file 1 [file marinedrugs-15-00243-s001.pdf]

Molecular identification of *Gambierdiscus* and *Fukuyoa* (Dinophyceae) from environmental samples

Kirsty F. Smith, Laura Biessy, Phoebe Argyle, Tom Trnski, Tuikolongahau Halafihi and Lesley Rhodes

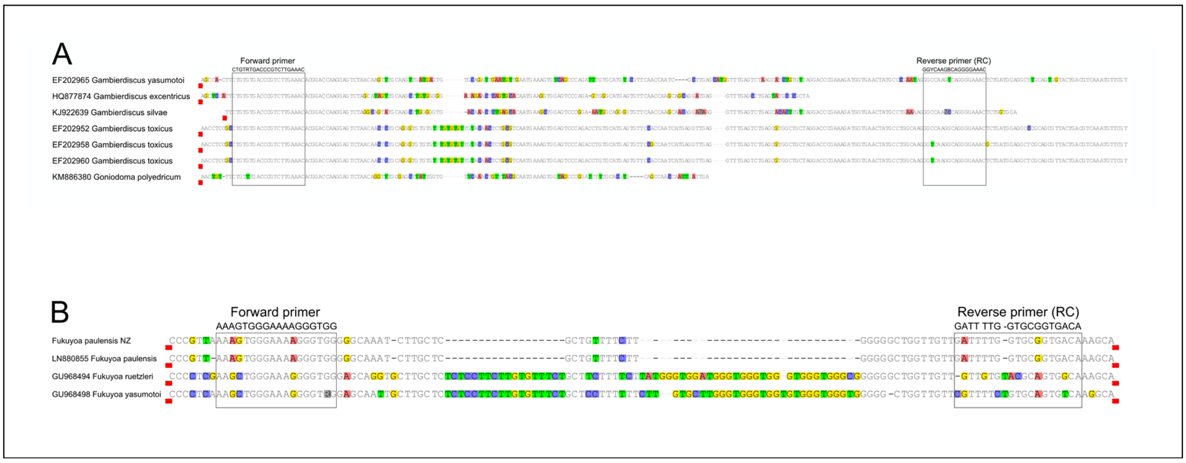

Figure S1: Alignments of available DNA sequences of *Gambierdiscus* and *Fukuyoa* species not able to be directly tested, and primers for both the (A) *Gambierdiscus/Fukuyoa* and (B) *Fukuyoa paulensis* QPCR assays.

Table S1. QPCR assay results for field samples collected in New Zealand and the Kingdom of Tonga. NT, not tested.

**Country: Site**	**Collection**	***Gambierdiscus/Fukuyoa***	***Fukuyoa paulensis***
**New Zealand**			
Oke Bay, Northland	Feb-14	-/-	NT
Site 1, Te Uenga Bay, Northland	Feb-14	-/-	NT
Site 2, Te Uenga Bay, Northland	Feb-14	+/+	+/+ (<1 cell)
Site 3, Te Uenga Bay, Northland	Feb-14	+/+	+/+ (<1 cell)
Site 4, Te Uenga Bay, Northland	Feb-14	-/-	NT
Site 1, Motuarohia Island, Northland	Feb-14	-/-	NT
Site 2, Motuarohia Island, Northland	Feb-14	-/-	NT
Site 1 at 12 m, North Meyer Island, Kermadec Is.	Nov-15	+/+	-/-
Site 2 at 17 m, North Meyer Island, Kermadec Is.	Nov-15	+/+	-/-
Site 3 at 17 m, North Meyer Island, Kermadec Is.	Nov-15	+/+	-/-
Site 4 at 13 m, North Meyer Island, Kermadec Is.	Nov-15	+/+	-/-
Site 5 at 12 m, North Meyer Island, Kermadec Is.	Nov-15	+/+	-/-
Site 6 at 13 m, North Meyer Island, Kermadec Is.	Nov-15	+/+	-/-
**Tongatapu, Kingdom of Tonga**			
Site 1, Kolonga	Jan-16	-/-	NT
Site 2, Kolonga	Jan-16	-/-	NT
Site 3, Kolonga	Jan-16	-/-	NT
Site 1, Lavengatonga	Jan-16	-/-	NT

Site 2, Lavengatonga	Jan-16	-/-	NT
Site 3, Lavengatonga	Jan-16	-/-	NT
Site 1, Ha'ateiho	Jan-16	+/+	-/-
Site 2, Ha'ateiho	Jan-16	-/-	NT
Site 3, Ha'ateiho	Jan-16	-/-	NT
Site 1, Sopu	Jan-16	+/+	-/-
Site 2, Sopu	Jan-16	+/+	-/-
Site 3, Sopu	Jan-16	-/-	NT
Site 4, Sopu	Jan-16	+/+	-/-
Site 5, Sopu	Jan-16	-/-	NT
Site 6, Sopu	Jan-16	-/-	NT
Site 1, Ha'atafu	Jan-16	+/+	-/-
Site 2, Ha'atafu	Jan-16	+/+	-/-
Site 3, Ha'atafu	Jan-16	-/-	NT

11

Table S2. Average read number and classification levels at 98% identity for the LSU gene region from each sampling site from North Meyer Island, Kermadec Islands, New Zealand.

**Species**	**Site 2**	**Site 3**	**Site 4**	**Site 5**	**Site 6**
Alexandrium sp.	0	11	5	1	1
Amphidinium massartii	3	12	4	3	2
Azadinium spinosum	4	0	3	1	0
Azadinium concinnum	22	13	13	11	12
Azadinium trinitatum	1	2	3	1	0
Biecheleria cincta	0	2	4	0	0
Dinophysis sp.	8	16	23	10	2
Gambierdiscus australes	12655	8817	7700	8769	8813
Gambierdiscus honu	56	77	111	3	25
Gambierdiscus polynesiensis	335	41	143	229	354
Heterocapsa triquetra	2	5	3	1	1
Lepidodinium sp.	13	39	31	10	4
Lepidodinium chlorophorum	3	2	5	2	1
Ostreopsis sp. 3	46	388	313	121	78
Prorocentrum hoffmannianum	2	38	34	1	2
Prorocentrum sculptile	7	4	6	3	1
Prorocentrum sp.	43	289	237	35	9
Scrippsiella sp.	5	15	22	9	5
Scrippsiella lachrymosa	4	7	8	2	0
Scrippsiella trochoidea	1	10	11	5	2
Symbiodinium sp. B	3	16	20	11	2
**Total number of Dinophyceae reads**	13210	9799	8693	9223	9309
**Number of Dinophyceae species**	19	20	21	20	17
